# NEDD4L Suppresses Proliferation and Promotes Apoptosis by Ubiquitinating RAC2 Expression and Acts as a Prognostic Biomarker in Clear Cell Renal Cell Carcinoma

**DOI:** 10.3390/ijms252211933

**Published:** 2024-11-06

**Authors:** Manlong Qi, Jianqiao Tu, Rong He, Xiang Fei, Yanyan Zhao

**Affiliations:** 1 Department of Clinical Genetics, The Affiliated Shengjing Hospital, China Medical University, Shenyang 110004, China; 18842485768@163.com (J.T.); her@sj-hospital.org (R.H.); 2 Department of Urology, The Affiliated Shengjing Hospital, China Medical University, Shenyang 110004, China; feix@sj-hospital.org

**Keywords:** renal cell carcinoma, ubiquitination, *NEDD4L*, *RAC2*

## Abstract

Neural precursor cell expressed developmentally down-regulated 4-like (NEDD4L) is an HECT (homologous to E6AP C terminus)-type E3 ubiquitin ligase. As previously documented, bioinformatics analysis revealed *NEDD4L* is downregulated in clear cell renal cell carcinoma (ccRCC). However, the target substrate regulated by NEDD4L in ccRCC remains unknown. Here, we assessed whether NEDD4L regulates Ras-related C3 botulinum toxin substrate 2 (RAC2) expression in ccRCC. In our study, integrated bioinformatics analysis indicated that low expression of *NEDD4L* and high expression of *RAC2* were both associated with poor prognosis of ccRCC, pro-tumorigenic immunity, and multiple tumor-associated pathways. Our data confirmed the hypothesis indicated in the previous studies related to the downregulation of *NEDD4L* in ccRCC. NEDD4L was identified to target the RAC2 threonine 108–proline motif, and *RAC2* overexpression rescued *NEDD4L*-mediated cell apoptosis and inhibition of cell growth and migration. Therefore, RAC2 is a novel and first identified target of NEDD4L in ccRCC, and the aberrant less expression of *NEDD4L* and consequent *RAC2* upregulation may contribute to renal carcinogenesis. Our study offers insight into *NEDD4L* as a potential future therapeutic target for renal cell carcinoma or as a novel prognostic biomarker.

## 1. Introduction

Renal cell carcinoma (RCC) is one of the most common malignant neoplasms in the genitourinary system, and RCC consists mainly of three major histological subtypes, clear cell RCC (ccRCC), papillary RCC (pRCC), and chromophobe RCC (chRCC). ccRCC is the most common and lethal form of urological malignancy diagnosed worldwide, accounting for ~75% of cases [1]. Epigenetic alterations, such as hypermethylation and histone modifications, have long been implicated in driving tumorigenesis in ccRCC [2,3,4,5]. Recent studies have shown that ubiquitination may play a critical role in RCC progression [6,7,8].

Ubiquitination is a three-step enzymatic cascade consisting of E1, E2, and E3 enzymes, and also a dynamic multifaceted post-translational modification involved in almost all aspects of eukaryotic biology and cellular responses to stress signals associated with cancer development [9,10]. The ubiquitination regulation is mainly accomplished by the binding of E3 ubiquitin ligases to particular targets and activating ubiquitin-mediated protein degradation. Therefore, E3 ligases play a pivotal role in ubiquitination modification and may be a promising target for cancer therapy.

E3 ligases either act as a scaffold to recruit an E2 enzyme and substrate (RING) or form a ubiquitin thioester intermediate before transferring ubiquitin to a substrate (HECT). The RING-inBetweenRING-RING (RBR) proteins are another specialized E3 ligase characterized by domain patterns of acidic residues (AC)-UBA-RING1-IBR-RING2-Ariadne using a hybrid RING/HECT mechanism to transfer ubiquitin to a substrate, and can be autoinhibited by the Ariadne domain through blocking the second RING-type zinc finger domain [11].

Neural precursor cell expressed developmentally down-regulated 4-like (NEDD4L) encodes a member of the NEDD4 family of HECT domain E3 ubiquitin ligases. *NEDD4L* is widely expressed in almost all tissues as two major isoforms with or without the C2 domain at the N-terminus, followed by conserved WW domains, and the HECT domain at the C-terminus. NEDD4L binds to substrates containing not only the canonical PY motif but also the serine/threonine–proline motif, and then transfers ubiquitin from E2 ubiquitin-conjugating enzymes to the ε-amino group of lysine residues on the substrates [12]. NEDD4L mediates the polyubiquitination of target proteins and is involved in the regulation of various signaling pathways including autophagy [13], innate immunity [14], DNA repair [15], ferroptosis [16], and EMT [17].

Diseases associated with *NEDD4L* include cardiovascular [18] and renal diseases [19], antiviral immunity [14], inflammatory responses [20], ophthalmic diseases [21], and seizure [22]. Pathogenic variants of *NEDD4L* can cause human paraventricular nodular heterotopia type 7 (PVNH7, OMIM # 617201), a neurological disorder characterized by abnormal neuronal migration during brain development with phenotypes of delayed psychomotor development, intellectual disability, and seizures [23]. Recently, more and more studies have focused on the role of *NEDD4L* in tumor pathogenesis. *NEDD4L* suppresses carcinogenesis by increasing substrate degradation in most tumor types, including gastric cancer [24], liver cancer [25], colorectal cancer [26], pancreatic cancer [27], lung cancer [28], ovarian cancer [29], endometrial cancer [30], and breast cancer [31], while the role of *NEDD4L* in gallbladder cancer [32] and prostate cancer [33,34,35] remains controversial. Bioinformatics analysis showed that low *NEDD4L* expression was associated with poor prognosis in patients with ccRCC and pRCC [36,37]. However, the target substrates regulated by *NEDD4L* in ccRCC and the exact molecular mechanism remain unclear.

In this study, we generated tubule-specific *NEDD4L* conditional knockout (CKO) mice. Renal proteomic analysis revealed potential substrates of NEDD4L, among which Ras-related C3 botulinum toxin substrate 2 (RAC2) was further confirmed as a novel substrate of NEDD4L through the threonine^108^–proline motif, and NEDD4L-mediated RAC2 upregulation was associated with ccRCC by comprehensive analysis and functional study.

## 2. Results

### 2.1. Generation of Renal NEDD4L CKO Mice

**Figure 1:**
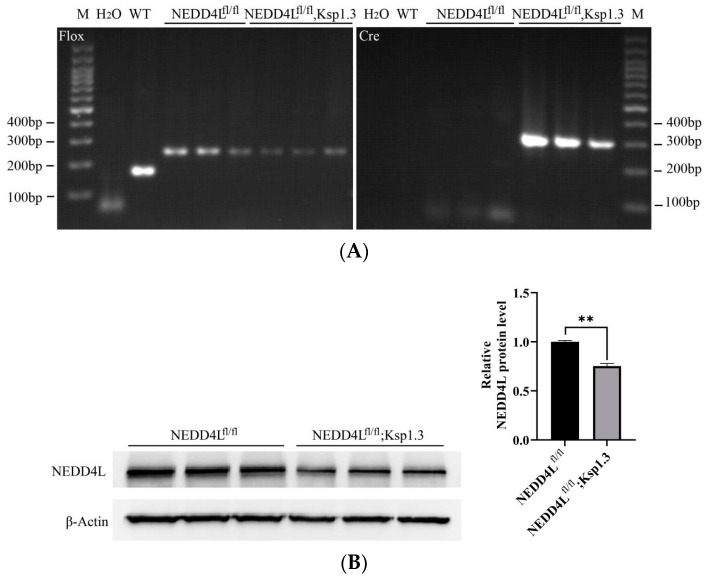
Identification of renal tubule-specific *NEDD4L* CKO mice. (**A**) Mouse genotyping by PCR using *NEDD4L*-CKO specific primers and general Cre genotyping primers. (**B**) NEDD4L protein assayed by Western blot in kidney tissue. ** *p* < 0.01.

Integrated bioinformatics data suggested the role of *NEDD4L* as a tumor suppressor in ccRCC [36,37]. In order to find potential targets of *NEDD4L* in the kidneys, we generated inducible renal tubule *NEDD4L* CKO mice as described previously [38]. The PCR genotyping results are shown in Figure 1A. Western blot on total kidney lysates revealed a decrease in the NEDD4L protein in NEDD4L^flox/flox^-Ksp1.3-Cre mice (Figure 1B).

### 2.2. Renal Proteomic Analysis in the NEDD4L CKO Mice

**Figure 2:**
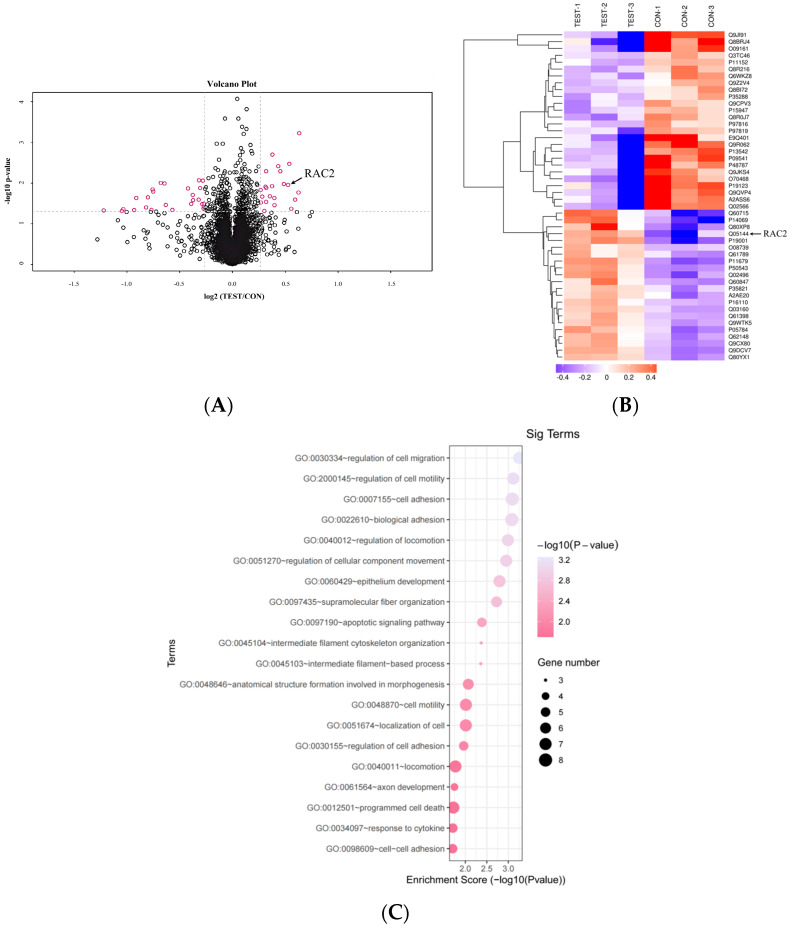
Renal proteomic analysis in the renal tubule-specific *NEDD4L* CKO mice. (**A**) Volcano plot and (**B**) heat map of proteins with increased (fold changes > 1.2) and decreased (fold changes < 0.83) abundance in renal NEDD4L^fl/fl^; Ksp1.3 over NEDD4L^fl/fl^ mice. (**C**) Top 20 Kyoto Encyclopedia of Genes and Genomes (KEGG) pathways of the altered proteins.

The total protein was obtained from the kidneys of NEDD4L^flox/flox^-Ksp1.3-Cre mice and control, and the proteomes were assayed using tandem mass tag (TMT) quantitative proteomics technology. In total, 5434 proteins were identified, and differentially expressed proteins (DEPs) were screened according to fold change (more than 1.2-fold or less than 0.83-fold) and *p* value < 0.05. A total of 48 significant genes were identified (22 upregulated genes and 26 downregulated genes) in the CKO TEST_vs_CON group (Figure 2A,B and Table 1). GO and KEGG pathway enrichment analysis revealed that the DEPs were mainly enriched in cell adhesion, cell migration, apoptosis, immune response, cell proliferation, and cell differentiation (Figure 2C, Table S1), indicating that *NEDD4L* may be involved in many cancer risk pathways. These pathways consisted of many proteins including P4HA1, RAC2, TNC, CYGB, NFKB2, LAMA3, and GRB7, which might be relevant to the protein trafficking and degradation function of NEDD4L.

### 2.3. RAC2 Is Upregulated and Is the Candidate Substrate of NEDD4L in ccRCC

**Figure 3:**
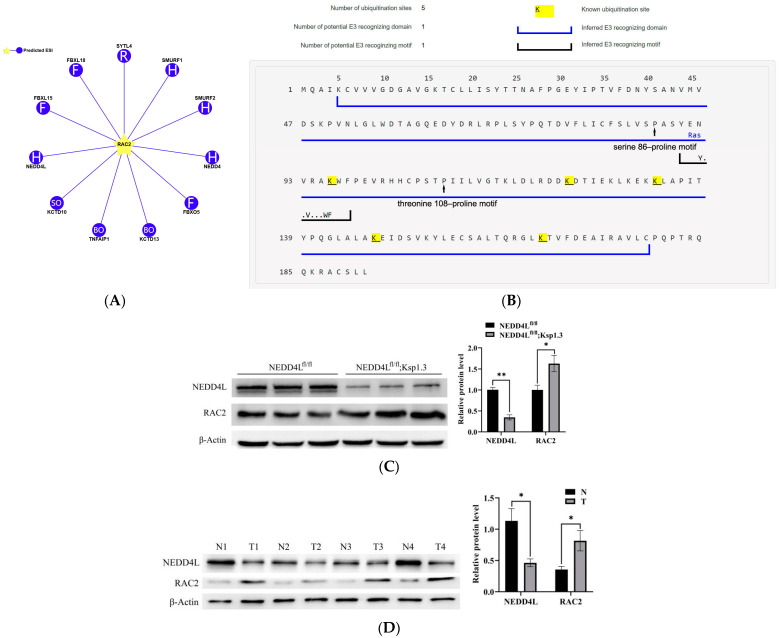

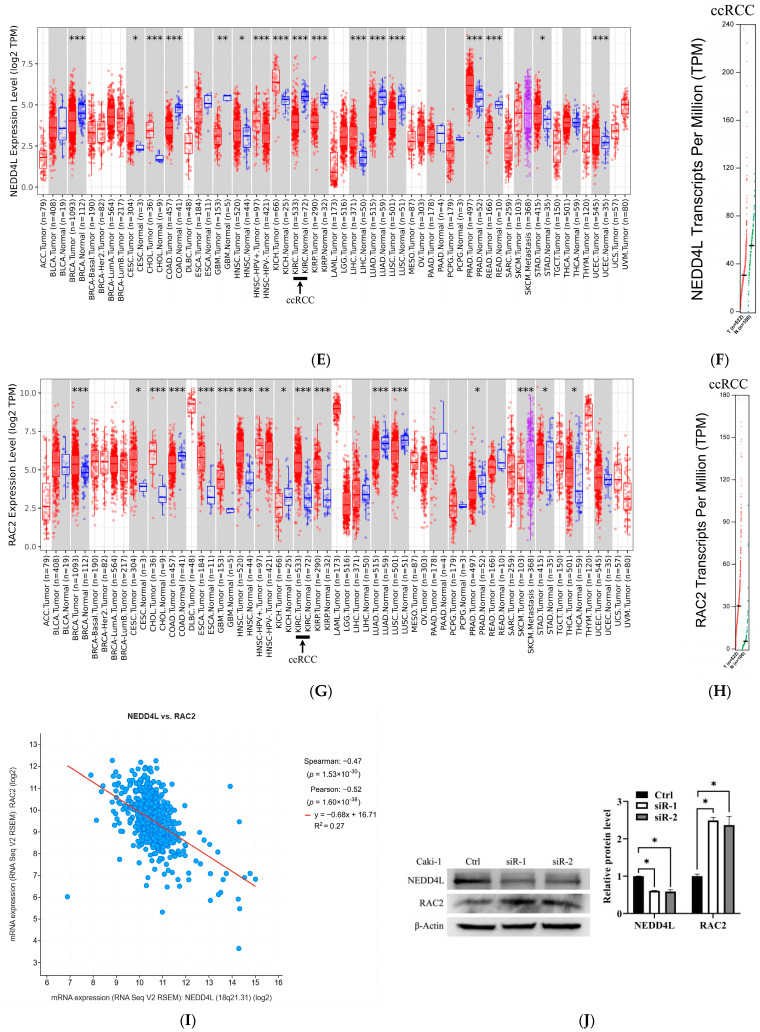
Expression levels of *NEDD4L* and *RAC2* in kidneys of *NEDD4L* CKO mice and ccRCC tissue. (**A**) E3 ubiquitin ligases of RAC2 predicted by UbiBrowser 2.0. (**B**) Known ubiquitination sites and inferred E3 recognizing domain and motif of RAC2. The predicted binding site of NEDD4L is indicated in the serine 86–proline motif and threonine 108–proline motif. (**C**) NEDD4L and RAC2 protein levels in NEDD4L^fl/fl^ and NEDD4L^fl/fl^; Ksp1.3 mice. (**D**) NEDD4L and RAC2 protein levels in ccRCC tumor (red) compared with paratumor tissues (blue). Downregulated *NEDD4L* expression in ccRCC analyzed by TIMER 2.0 (**E**) and GEPIA2 (**F**). Upregulated *RAC2* expression in ccRCC using TIMER 2.0 (**G**) and GEPIA2 database (**H**). (**I**) *NEDD4L* was negatively correlated with *RAC2* mRNA expression using cBioPortal database. (**J**) *NEDD4L* siRNA mediated inhibition of *NEDD4L* induced upregulation of RAC2 expression. * *p* < 0.05, ** *p* < 0.01, *** *p* < 0.001.

Proteomic analysis revealed a 1.44-fold increase in RAC2 expression in *NEDD4L* CKO mice (*p* = 0.011043). Among the proteins in the enriched pathways, *RAC2* is the only high-confidence substrate of NEDD4L predicted by UbiBrowser 2.0 with a confidence score of 0.813 and likelihood ratio of 29.29 (Figure 3A,B). Therefore, we selected *RAC2* for further studies to analyze the role of *NEDD4L* in ccRCC. To confirm this prediction, we detected the levels of RAC2 and NEDD4L in CKO mice kidneys and ccRCC tumors by Western blot analysis. As shown in Figure 3C,D, RAC2 expression was significantly upregulated and NEDD4L expression was downregulated in CKO mice kidneys and ccRCC tumors compared with those in the WT controls and paratumor tissues. Online TCGA data analysis also confirmed the low expression of *NEDD4L* (Figure 3E,F) and high expression of *RAC2* (Figure 3G,H) in ccRCC using TIMER 2.0 and GEPIA2, while the cBioPortal database further revealed the inverse correlation between *RAC2* and *NEDD4L* expression (Figure 3I). Furthermore, *NEDD4L* knockdown by *NEDD4L* siRNA markedly increased the endogenous RAC2 protein expression of Caki-1 cells (Figure 3J, Table S2). We concluded that low levels of *NEDD4L* may contribute to the upregulation of *RAC2* in ccRCC.

### 2.4. NEDD4L Interacted with RAC2 Through the Threonine^108^–Proline Motif

**Figure 4:**
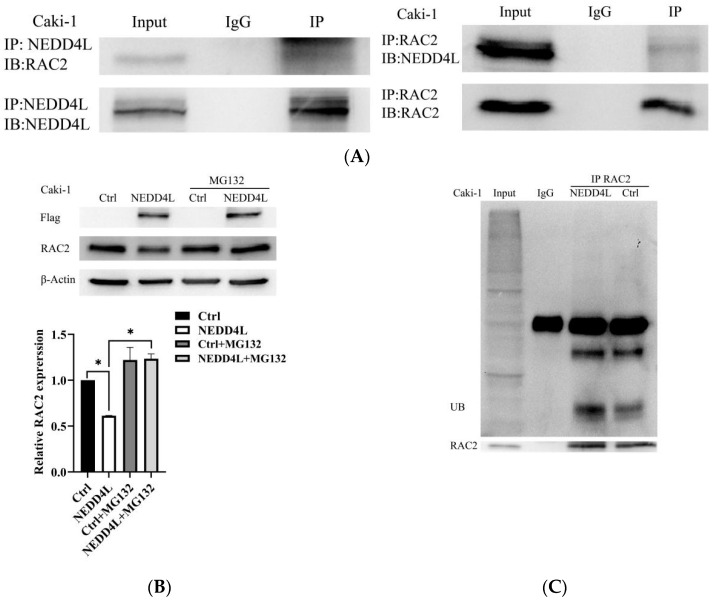

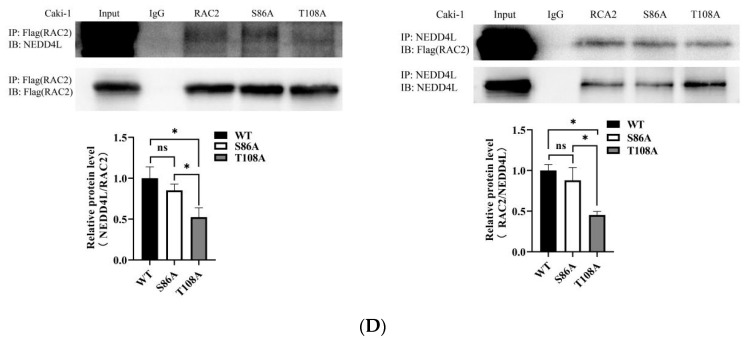
RAC2 is ubiquitinated by NEDD4L through the threonine 108–proline motif. (**A**) Co-immunoprecipitation (IP) analysis for the interaction of NEDD4L and RAC2 in Caki-1 cells. (**B**) RAC2 expression in Caki-1 cells transfected with *NEDD4L*-overexpressing plasmid (NEDD4L) or control plasmid (Ctrl) in the presence of 20 µM MG132 or control, β-Actin as the loading control. (**C**) *NEDD4L*-overexpressed Caki-1 cells immune-precipitated with RAC2 or IgG antibody for evaluating ubiquitination. (**D**) RAC2 mutant T108A at the threonine108–proline motif abolished the interaction with NEDD4L. ns: not significant. * *p* < 0.05.

NEDD4L-mediated ubiquitination of RAC2 was testified by co-IP in Caki-1 cells. Endogenous NEDD4L successfully pulled down endogenous RAC2, and vice versa (Figure 4A). To investigate the potential ubiquitination of RAC2 by NEDD4L, we treated the cells with the proteasome inhibitor MG132. The downregulated protein expression of RAC2 caused by *NEDD4L* overexpression in Caki-1 cells could be rescued by MG132 treatment (Figure 4B), suggesting that NEDD4L regulates RAC2 levels in a proteasome-dependent manner. Moreover, *NEDD4L* overexpression in Caki-1 cells strengthened the ubiquitination of RAC2 compared to that of the control cells (Figure 4C). All these data indicated that RAC2 may be a novel substrate of NEDD4L in ccRCC.

To identify the critical binding motif of RAC2 with NEDD4L, we constructed two RAC2 mutants with S86A and T108A at the putative serine/threonine–proline motifs. These Flag-tagged mutant or WT *RAC2* vectors were transfected to Caki-1 cells. In the co-IP experiments (Figure 4D), the interaction with NEDD4L was attenuated to about 20% of the WT level when Caki-1 was mutated at T108A. The S86A mutant hardly affected the interaction. Thus, the threonine108–proline motif of RAC2 was critical for the interaction with NEDD4L.

### 2.5. High RAC2 Expression and Low NEDD4L Expression Were Correlated with Poor Prognosis in ccRCC Patients

**Figure 5:**
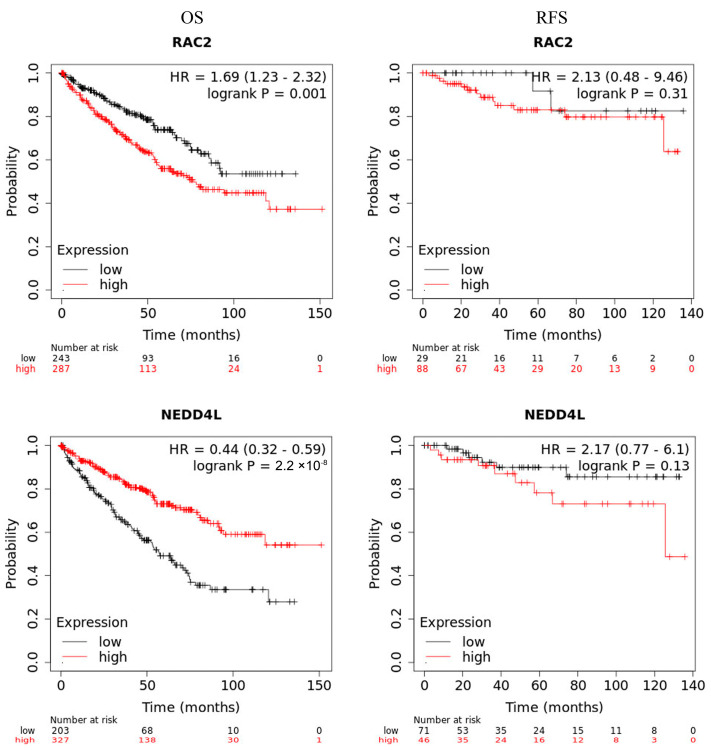
Survival analysis in TCGA ccRCC. Kaplan–Meier curves represent *RAC2* or *NEDD4L* association with patient overall survival and relapse-free survival in ccRCC.

The association between *RAC2* and *NEDD4L* expression and the clinicopathological characteristics including age, sex, grade, TNM stage, invasion depth, lymph node metastasis, distant metastasis, and vital status of ccRCC patients was investigated using TCGA data through UCSC Xena tools. According to the median values of RAC2 or NEDD4L mRNA level, 523 patients were divided into high and low expression groups. As shown in Table 2, *RAC2* expression was significantly positively correlated with grade (<0.001), TNM stage (*p* = 0.014), lymph node metastasis (*p* = 0.004), distant metastasis (*p* = 0.034), and vital status (*p* = 0.003), while *NEDD4L* expression was significantly negatively correlated with grade (<0.001), TNM stage (*p* < 0.001), invasive depth (*p* < 0.001), distant metastasis (*p* = 0.001), and vital status (*p* < 0.001) in ccRCC patients. Univariate and multivariate analysis were used to validate the prognostic value of *RAC2* and *NEDD4L* in ccRCC. The results of the univariate analysis demonstrated that high *RAC2* and low *NEDD4L* expression were associated with worse overall survival (OS) (hazard ratio [HR]: 1.442; 95% CI [1.061–1.958]; *p* = 0.019 and hazard ratio [HR]: 0.506; 95% CI [0.370–0.691]; *p* < 0.001, respectively). Multivariate COX regression analysis revealed that low expression of *NEDD4L* was still an independent factor associated with the deterioration in OS (HR: 0.839; CI [0.709–0.993], *p* = 0.041) (Table 3). The Kaplan–Meier plotter also confirmed that high expression of RAC2 and low expression of NEDD4L were both significantly associated with shorter overall survival (HR = 1.69, *p* = 0.001 and HR = 0.44, *p* = 2.2 × 10^−8^, respectively), although neither was associated with relapse-free survival (RFS) (Figure 5). These results suggested that high expression of *RAC2* and low expression of *NEDD4L* were correlated with dismal prognosis in ccRCC patients.

### 2.6. RAC2 Was Positively and NEDD4L Was Negatively Correlated with Tumor-Infiltrating Immune Cells in ccRCC

**Figure 6:**
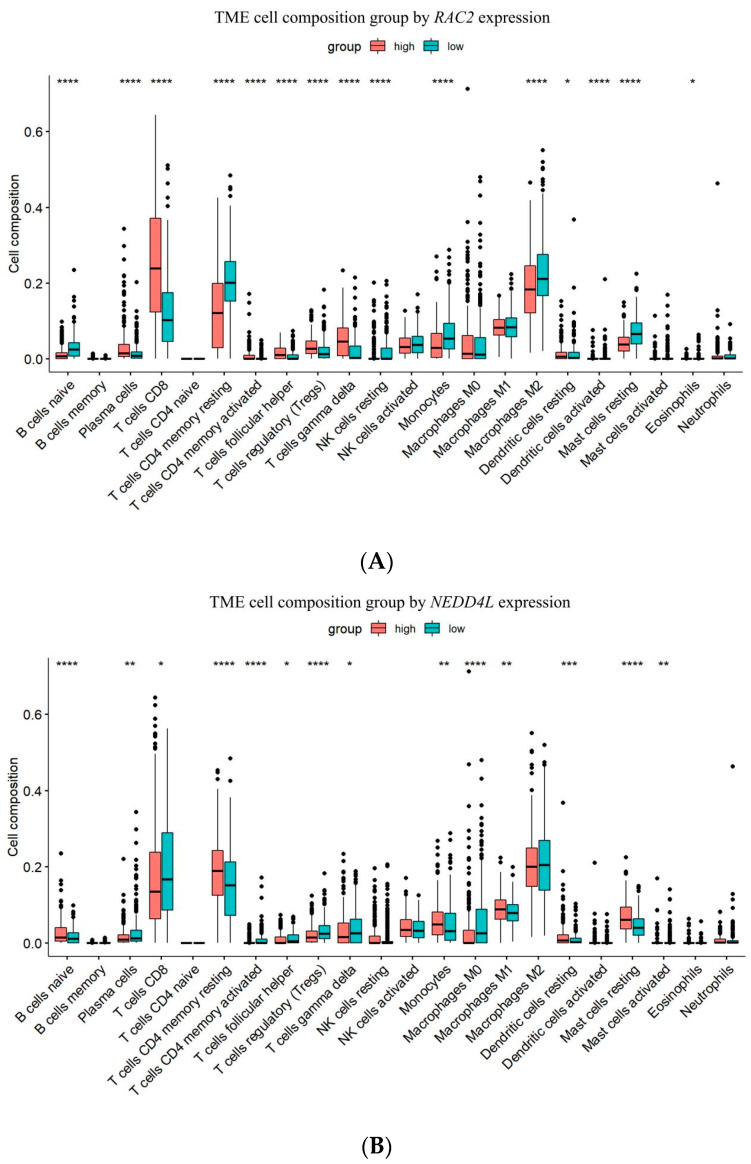

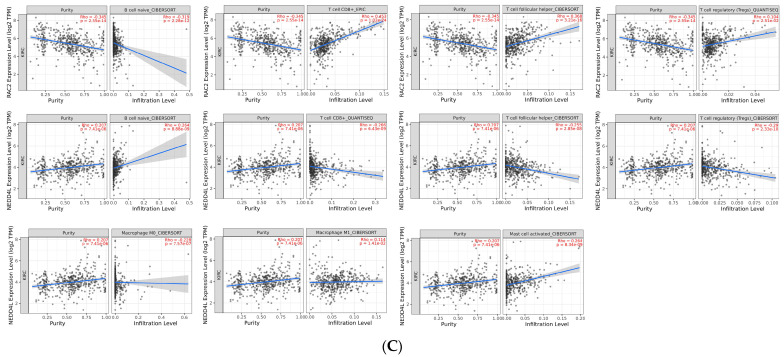
Correlations between expression level of *RAC2* or *NEDD4L* and the infiltration levels of immune cells. The influence of *RAC2* (**A**) or *NEDD4L* (**B**) expression on the infiltrated patterns of 22 immune cells in ccRCC using CIBERSORT. (**C**) The relationship between *RAC2* or *NEDD4L* expression and the infiltration level of B cell naive, T cell CD8+, T cell follicular helper, Tregs, macrophage M0, M1, and mast cell activated using TIMER 2.0. * *p* < 0.05, ** *p* < 0.01, *** *p* < 0.001, **** *p* < 0.0001.

The influence of *RAC2* and *NEDD4L* expression on the infiltration patterns of 22 immune cells in ccRCC was estimated using CIBERSORT R script v1.03. The results showed that *RAC2* expression was significantly positively correlated with the levels of tumor-filtrating immune cells, including CD8+ T cells (*p* < 0.0001), follicular helper T cells (*p* < 0.0001), and regulatory T cells (Tregs) (*p* < 0.0001), and negatively correlated with naive B cells and resting mast cells (*p* < 0.0001) (Figure 6A). On the contrary, *NEDD4L* expression was positively correlated with naive B cells (*p* < 0.0001), M1 macrophages (*p* = 0.0033), and resting mast cells (*p* < 0.0001), and negatively correlated with the ratio of CD8+ T cells (*p* = 0.016), follicular helper T cells (*p* < 0.0001), Tregs (*p* < 0.0001), M0 macrophages (*p* < 0.0001), and activated mast cells (*p* = 0.0028), which have tumor-promoting effects (Figure 6B). Similar results were also achieved using the TIMER 2.0 database (Figure 6C). These data suggested that *RAC2* may be involved in the immunosuppressive response in the tumor immune microenvironment of ccRCC; conversely, *NEDD4L* may inhibit tumorigenesis by promoting anti-tumor immunity.

### 2.7. Gene Function and Pathway Analysis of RAC2 and NEDD4L in the ccRCC Dataset

**Figure 7:**
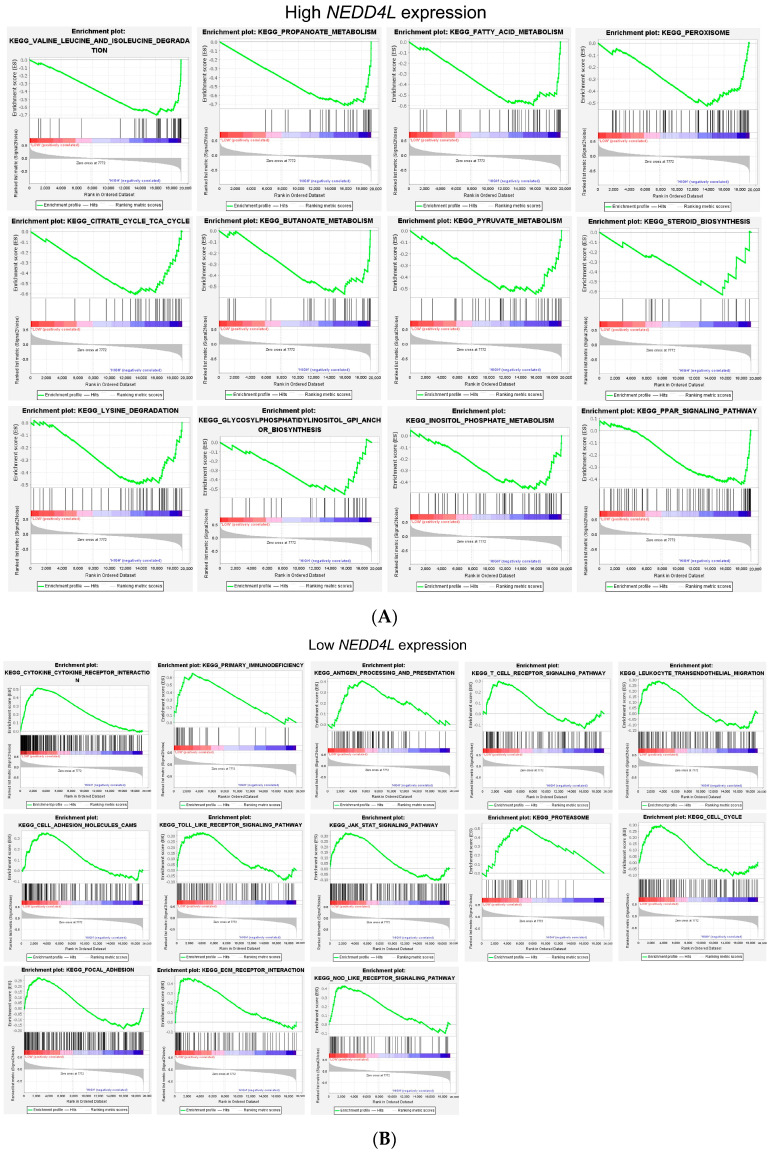

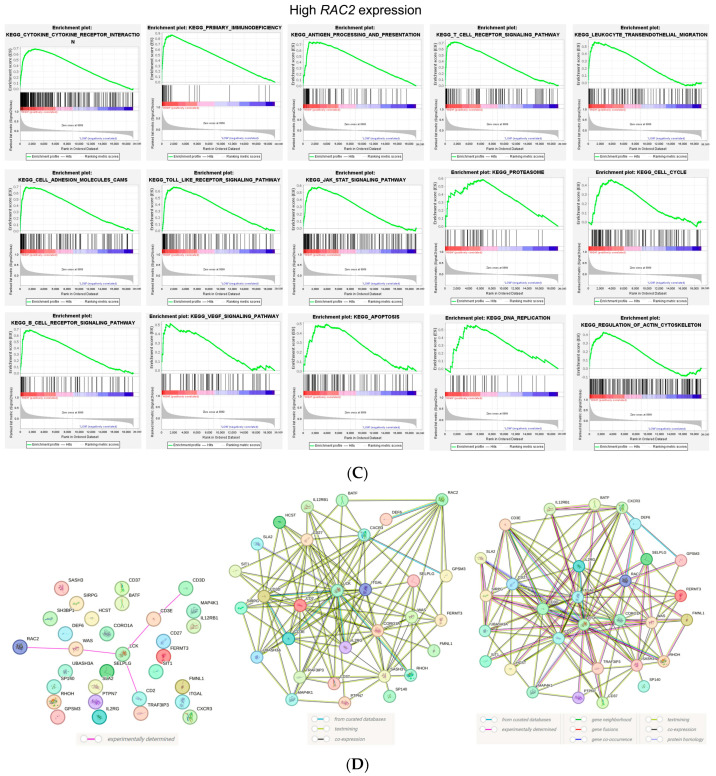
GSEA analysis of *RAC2* or *NEDD4L* co-expression genes in TCGA-KIRC samples. GSEA between ccRCC patients with high *RAC2* (**A**) or low *NEDD4L* (**B**) or high *NEDD4L* (**C**) expression. “Low”: Low level of *NEDD4L* or *RAC2* expression; “High”: High level of *NEDD4L* or *RAC2* expression. (**D**) Protein and protein interaction networks analysis for RAC2 using STRING. The network plot including experimentally supported interactions, or curated database, text mining, and co-expression supported interactions, or all supported interactions were listed accordingly.

The tumorigenesis of *RAC2* and *NEDD4L* in the occurrence of ccRCC is not clear. Considering the upregulated *RAC2* expression and downregulated *NEDD4L* expression in ccRCC, we performed TCGA_ccRCC GSEA (Version: 4.2.3) to find possible activated signal pathways in the abnormal expression of *RAC2* or *NEDD4L* in ccRCC. According to the normalized enrichment score (NES), the signaling pathways most significantly associated with *RAC2* and *NEDD4L* expression are shown in Figure 7A–C and Table 4, Table 5 and Table 6. The analysis results showed that *NEDD4L* expression was positively correlated with many pathways, such as propanoate, fatty acid, butanoate, pyruvate, inositol phosphate metabolism, valine, leucine, isoleucine and lysine degradation, steroid biosynthesis and glycosylphosphatidylinositol/GPI anchor, peroxisome, citrate cycle/TCA, and PPAR signaling pathways, which were downregulated in ccRCC. Interestingly, low expression of *NEDD4L* and high expression of *RAC2* shared many similar tumor-associated pathways including immune regulation (cytokine–cytokine receptor interaction, primary immunodeficiency, antigen processing and presentation, T cell receptor signaling pathway, and leukocyte transendothelial migration), proteasome pathway, cell adhesion, cell cycle, Toll-like receptor signaling pathway, and JAK-STAT signaling pathway. In addition, low expression of *NEDD4L* was also positively correlated with ECM receptor interaction, the Nod-like receptor signaling pathway, and high expression of *RAC2* was positively correlated with the B cell receptor signaling pathway, VEGF signaling pathway, apoptosis, actin cytoskeleton regulation, and DNA replication. We also analyzed the 30 most frequently altered neighbor genes (Table S3) of *RAC2* using the cBioPortal online tool (https://www.cbioportal.org/, accessed on 21 March 2024) for kidney renal clear cell carcinoma (TCGA, Firehose Legacy), and constructed their interaction network using STRING version 12.0 (Figure 7D). *WAS*, a member of the Wiskott–Aldrich syndrome protein (WASP) family, including *WASF1* and *WASF2*, is the only experimentally supported interactor of *RAC2* [39]. The functions and pathways of *RAC2* and the associated genes were similar to those in the GSEA analysis, including immune regulation (primary immunodeficiency, *PD-L1* expression, and *PD-1* checkpoint pathway in cancer, T cell receptor signaling pathway, and cytokine–cytokine receptor interaction) and cell adhesion (Table S4). These results indicated that *RAC2* and *NEDD4L* may participate in tumor immune regulation and affect the proteasome pathway, cell growth, adhesion, and apoptosis.

### 2.8. NEDD4L Suppresses Proliferation and Promotes Apoptosis by Suppressing RAC2 Expression In Vitro

**Figure 8:**
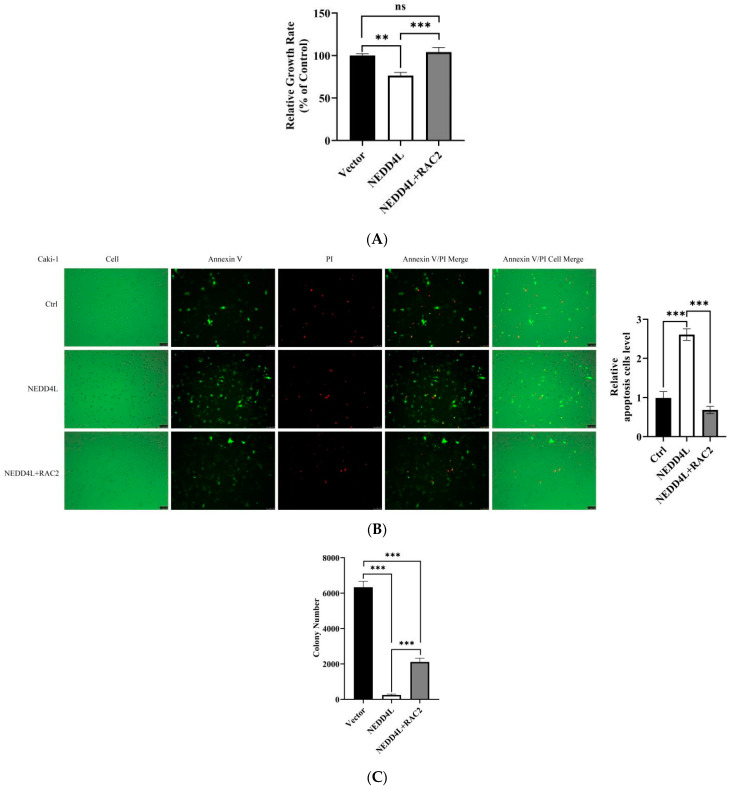

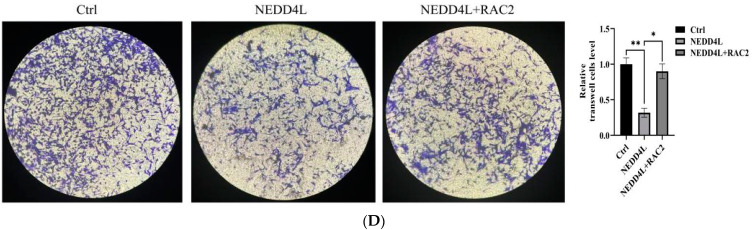
*RAC2* inhibited *NEDD4L*-mediated anti-renal cancer function. Caki-1 cells were transfected with *RAC2*-overexpressing plasmid (RAC2) after *NEDD4L*-overexpressing plasmid or control transfection. (**A**) Cell viability assay by MTS. (**B**) Cell apoptosis assessed by fluorescence detection of Annexin V-AbFlourTM 488/Propidium Iodide (PI) (20× magnification). (**C**) Tumorigenesis potential of Caki-1 cells was determined using soft agar colony formation assay. (**D**) Cell migration assay was performed using Transwell chamber (10× magnification). ns: not significant. * *p* < 0.05, ** *p* < 0.01, *** *p* < 0.001.

We next evaluated the effect of NEDD4L-mediated RAC2 ubiquitination on tumor cell growth. We first examined the proliferation rates of Caki-1 cells transiently transfected with *NEDD4L* expression vectors. As shown in Figure 8A, the *NEDD4L* expression vector-transfected cells exhibited significant growth suppression compared to the NC-transfected or the untreated Caki-1 cells, and *RAC2* expression vector co-transfected cells could rescue the growth of Caki-1 cells at 48 h post-transfection. Moreover, exogenous *RAC2* overexpression also significantly inhibited *NEDD4L*-induced apoptosis of Caki-1 cells compared to the control cells at 48 h post-transfection (Figure 8B). These results suggested that *NEDD4L*-mediated growth inhibition and cell apoptosis in Caki-1 cells may occur in an *RAC2*-dependent manner.

To determine the role of NEDD4L-mediated RAC2 ubiquitination in renal tumorigenesis, we next carried out soft agar colony formation assays and cell migration of Caki-1 cells transfected with *RAC2*-overexpressing plasmid (RAC2) after *NEDD4L*-overexpressing plasmid or control plasmid. As shown in Figure 8C,D, the *NEDD4L* expression vector-transfected cells formed fewer colonies and suppressed the cell migration of Caki-1 cells compared to the NC-transfected cells; however, exogenous *RAC2* overexpression could effectively rescue the colony formation and cell migration, indicating that *NEDD4L* may inhibit the tumorigenicity of renal cancer cells through the suppression of *RAC2* expression.

### 2.9. NEDD4L Activates Apoptosis Signaling Pathway by Suppressing RAC2 Expression

**Figure 9:**
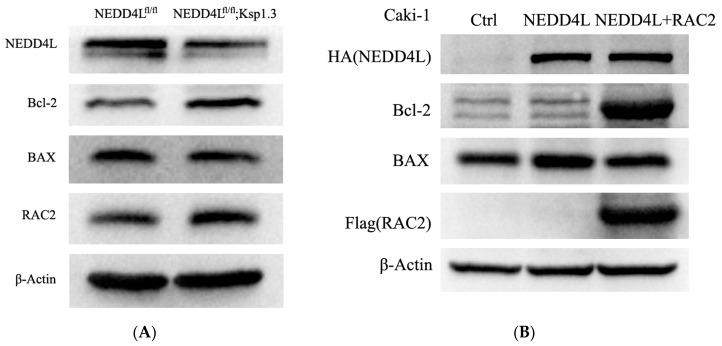
*RAC2* inhibited *NEDD4L*-mediated Bax/Bcl-2 apoptosis signaling pathway. (**A**) Bcl-2 and BAX protein levels in NEDD4L^fl/fl^ and NEDD4L^fl/fl^; Ksp1.3 mice. (**B**) Caki-1 cells were transfected with *RAC2*-overexpressing plasmid (RAC2) after *NEDD4L*-overexpressing plasmid or control transfection. Bcl-2 and BAX protein levels were detected at 48 h post-transfection.

We performed Western blot to detect the levels of apoptosis-related proteins in CKO mice kidneys and Caki-1 cells. As shown in Figure 9A, the ratio of Bax/Bcl-2 was significantly downregulated in CKO mice kidneys compared with those in the WT controls. The *NEDD4L* expression vector-transfected cells exhibited a higher Bax/Bcl-2 ratio than the NC-transfected Caki-1 cells, and the *RAC2* expression vector co-transfected cells could rescue the Bax/Bcl-2 ratio at 48 h post-transfection (Figure 9B). These results suggested *NEDD4L*-mediated Bax/Bcl-2 apoptosis signaling pathway activation may occur in an *RAC2*-dependent manner

## 3. Discussion

A recent research hotspot in ccRCC has focused on the role of ubiquitination in tumorigenesis. *NEDD4L* is an important E3 ubiquitin ligase that mediates the polyubiquitination of lysine and cysteine residues on target proteins. *NEDD4L* CKO mice proteomics analysis revealed the majority of the 22 genes upregulated were involved in tumor-associated signaling pathways, with *GRB7*, *LGALS3*, *LAMA3*, *RAC2*, *TNC*, *CYGB,* and *S100A11* involved in the regulation of cell migration, cell motility, and cell localization, and *LGALS3*, *MUC1*, *KRT18*, *LAMA3*, *COL12A1*, *RAC2*, *TNC,* and *S100A11* in cell adhesion, and *KRT19*, *KRT18,* and *KRT7* in intermediate filament cytoskeleton organization. In addition, *LAMA3*, *S100A6*, *RAC2,* and *TNC* are involved in axon development related with neural development and *PTPN1*, *KRT18*, *ALDH1A2*, KRT8, and *NFKB2* are involved in immune responses. All of these pathways are relevant to the diseases including tumors, inflammation, and neuronal developmental abnormalities caused by *NEDD4L*. Similar to previous reports [36,37,40], our study revealed that *NEDD4L* was downregulated and *RAC2* was upregulated in ccRCC tissues, and low expression of *NEDD4L* and high expression of *RAC2* were both associated with poor prognosis, shorter survival, higher tumor–node–metastasis stage, and worse G grade. Low *NEDD4L* expression was an independent risk factor for ccRCC. Importantly, our data provided evidence for the first time from *NEDD4L* CKO mice and renal cancer Caki-1 cells supporting RAC2 as a novel ubiquitylation target of NEDD4L in ccRCC. RAC2 interacted with NEDD4L through the threonine^108^–proline motif. In *NEDD4L* CKO mice and ccRCC tissues, *NEDD4L* expression was inversely correlated with *RAC2* expression, and NEDD4L directly reduced RAC2 protein levels, suggesting that aberrant *NEDD4L* expression may be a novel mechanism underlying *RAC2* upregulation in renal cancer. We also demonstrated that NEDD4L inhibited the growth and migration of Caki-1 cells by ubiquitinating RAC2, and *RAC2* overexpression could rescue *NEDD4L*-induced cell growth and migration inhibition and reduce *NEDD4L*-induced cell apoptosis. Pathway enrichment analysis revealed that low expression of *NEDD4L* and high expression of *RAC2* shared many similar tumor-associated pathways, including immune regulation, the proteasome pathway, cell adhesion, cell cycle, the Toll-like receptor signaling pathway, the JAK-STAT signaling pathway, and leukocyte transendothelial migration. These findings suggested that NEDD4L may be a tumor suppressor that ubiquitinates and degrades RAC2 in ccRCC.

*NEDD4L* acts as an oncogene and is expressed at low levels in tumors such as breast cancer [31], colon adenocarcinoma [41], glioma [42], esophageal cancer [43], lung cancer [28], hepatocellular cancer [25], and endometrial cancer [30], as it does in ccRCC. Previous studies have shown that *NEDD4L* can inhibit TGF-beta signaling by Smad2/3 ubiquitination in mouse embryonic stem cells [44], downregulate autophagy and cell growth by ubiquitinating and reducing cellular ULK1 levels in cervical cancer cells [45], deceased pancreatic cancer cell proliferation and metastasis by ANXA2 ubiquitination [46] and inhibit glycolysis and promote apoptosis by ubiquitinating STK35 in colorectal cancer [41], ubiquitylate 8-oxoguanine DNA glycosylase (OGG1) to modulate the cellular DNA damage response [47], catalyze Dishevelled 2 (Dvl2) polyubiquitination to inhibit Wnt signaling [48], and mediate the ubiquitination of PIK3CA to weaken PI3K-AKT signaling [49]. However, our bioinformatics analysis of the TCGA database also showed that *NEDD4L* is highly expressed in cervical cancer, cholangiocarcinoma, head and neck squamous cell carcinoma, and renal chromophobe, and there is no difference in *NEDD4L* expression in adrenocortical carcinoma, pancreatic adenocarcinoma, pheochromocytoma, thyroid cancer, and thymoma compared to normal tissue. The mechanism and clinical significance of high *NEDD4L* expression in the above tumors are unclear.

So far, only two papers have been reported on the role of NEDD4L in ccRCC [36,37], mainly focusing on bioinformatics studies. Consistent with previous studies, our analysis suggested that *NEDD4L* expression might be associated with cell apoptosis and immune response. Bioinformatics analysis provided some novel functional evidence for *NEDD4L* in ccRCC, indicating that *NEDD4L* is involved in many metabolic pathways, including the propanoate, fatty acid, butanoate, pyruvate, inositol phosphate metabolism, valine, leucine, isoleucine and lysine degradation, steroid and glycosylphosphatidylinositol/GPI anchor biosynthesis, peroxisome, citrate cycle/TCA, and PPAR signaling pathway. Furthermore, in the immune microenvironment, *NEDD4L* can promote the infiltration of naive B cells, M1 macrophages, and resting mast cells, and decrease the proportion of follicular helper T cells (Tfh), regulatory T cells (Tregs), M0 macrophages, and activated mast cells. A low level of *NEDD4L* expression can increase ECM receptor interaction, promote cell adhesion, and the cell cycle. None of the above has been previously reported. Naive-like B cells have been reported to suppress the growth of lung cancer cells and are associated with a good prognosis in non-small cell lung cancer [50]. M1 macrophages can directly kill tumor cells and are effective in cancer-targeted therapies [51,52]. Resting mast cells were associated with favorable prognosis, whereas Tfh, Tregs, and M0 macrophages were correlated with worse outcomes in ccRCC [52,53]. Therefore, we concluded that downregulated *NEDD4L* in ccRCC might lead to metabolic disorder and immune deficiency, inhibit cell apoptosis, promote cell growth, migration, and adhesion, and further induce the development of ccRCC.

*RAC2* is the first identified target of *NEDD4L* in renal cell carcinoma. *RAC2* is a member of the Ras superfamily of small guanosine triphosphate (GTP)-metabolizing proteins. Activated RAC2 binds to a variety of effector proteins to regulate cellular responses, such as phagocytosis of apoptotic cells [54,55], epithelial cell polarization [56], and the production of reactive oxygen species (ROS) by NADPH oxidase [57]. Recent studies have suggested that *RAC2* acts inconsistently in different tumors. *RAC2* expression was significantly downregulated in breast cancer [58,59]. However, Pei et al. [60] found that *RAC2* was upregulated in non-small cell lung cancer and was associated with poor prognosis. Higher expression levels of *RAC2* were associated with higher clinical and pathological grades and poorer OS in patients with ccRCC [40], but the underlying molecular mechanism was not clear. Few studies have focused on the ubiquitination modification of RAC2. HACE1 was the only reported E3 ligase of RAC2 [51,61]. In our study, RAC2 acts as the substrate of NEDD4L, and a low level of *NEDD4L* was attributed to the upregulation of RAC2 expression. Increased *RAC2* expression further promoted cell growth and migration, and decreased cell apoptosis. Bioinformatics suggested that *RAC2* may be involved in cell adhesion, the VEGF signaling pathway, apoptosis, cell cycle, DNA replication, and immune regulation through possible interactions with *CD3D*, *IL2RG*, *LCK*, *BATF*, *CXCR3,* and *ITGAL*, promoted the infiltration of CD8+ T cells, Tfh, and Tregs, leading to the development and progression of tumors. Most of the above pathways have not been mentioned before and need further investigation.

Notably, our analysis data revealed that *WAS* is the only experimentally confirmed interactor of *RAC2*. *WASF1* plays a critical role downstream of Rac in the regulation of the actin cytoskeleton required for membrane ruffling. Diseases associated with *WASF1* include neurodevelopmental disorder with absence of speech and variable seizures and non-specific syndromic intellectual disability [62]. The *NEDD4L*/*RAC2/WASF1* may be a novel pathway associated with *NEDD4L*-related neurodevelopmental and intellectual disabilities. Moreover, the germline pathogenic mutation of *NEDD4L* can lead to PVNH7. Seven of the eight known mutations occur in the HECT domain [23]. HECT mutations may induce constitutive activation leading to NEDD4L autoubiquitination and sequential ubiquitination abnormalities of substrates [63,64]. Although a tumor is a somatic disease, HECT mutations might be a novel mechanism for the downregulation of *NEDD4L* expression in ccRCC and also deserve further exploration.

NEDD4L is an important E3 ligase involved in many diseases. Differences in clinical phenotypes may be due to differences in the expression patterns of *NEDD4L* and its ubiquitylated substrates in different tissues. In our study, we mainly focused on the mechanism of NEDD4L ubiquitylation of RAC2 in ccRCC, which is still a limitation for studying the mechanism of renal carcinogenesis. Finding more valuable substrates through bioinformatics and molecular biology techniques is necessary in *NEDD4L* studies.

## 4. Materials and Methods

### 4.1. Generation of Renal Tubule-Specific NEDD4L CKO Mice

All animal experiments were performed in accordance with protocols approved by the Institutional Ethics Committee of Shengjing Hospital of China Medical University. NEDD4L^flox/flox^ mice were obtained from the Shanghai Biomodel Organism Science & Technology Development Co., Ltd. (Shanghai, China). Inducible renal tubule NEDD4L^flox/flox^-Ksp1.3-Cre mice, referred to as NEDD4L^fl/fl^; Ksp1.3 mice, were generated as described previously [38]. Mice were genotyped by PCR using *NEDD4L*-CKO specific primers (TGATTGGAGTTATTCTGCGCCTCT and GTAAAGACATTGAAGCAAGCCAGT) and general Cre genotyping primers (CAGCATTGCTGTCACTTGGTC and ATTTGCCTGCATTACCGGTCG).

### 4.2. Renal Proteomic Analysis

Total protein was obtained from kidneys of renal NEDD4L^fl/fl^; Ksp1.3 and NEDD4L^fl/fl^ mice, and proteomics analysis was performed using renal total protein extracts (n = 9 for each group) by Shanghai Applied Protein Technology Co., Ltd. (Shanghai, China). The proteomics data were obtained and arbitrary fold change (FC) cut-offs of >1.2 and significance *p*-values of <0.05 were used to collect the differentially expressed genes. Volcano plots and heat maps were performed using R version 4.1.2. Pathway enrichment analysis and Kyoto Encyclopedia of Genes and Genomes (KEGG) were carried out using DAVID (https://david.ncifcrf.gov/, accessed on 2 March 2024). UbiBrowser 2.0 (http://ubibrowser.bio-it.cn/ubibrowser_v3/, accessed on 6 March 2024) was used to predict the ubiquitination substrates of NEDD4L.

### 4.3. Plasmid Construction and Cell Transfection

The human Flag-tagged *NEDD4L* (NM_015277), human HA-tagged *NEDD4L* (NM_015277), human Flag-tagged *RAC2* (NM_002872), and its two mutants’ (S86A and T108A) expression vectors were constructed by Shanghai GeneChem Co., Ltd. (Shanghai, China). The human *NEDD4L* small interfering RNA (siRNA) oligos (Table S1) were synthesized by JinXu Co., Ltd., (Nantong, China). ccRCC cell line Caki-1 cells (Cell Resource Center, Shanghai Institutes for Biological Sciences, Chinese Academy of Sciences, China) were cultured in McCoy’s 5A (Modified) medium (Bionlogical Industries, Kibbutz Beit, HaEmek, Israel) supplemented with 10% (*v*/*v*) fetal bovine serum (Sigma, St. Louis, MO, USA) and 1% penicillin/streptomycin (Thermo Fisher Scientific, Waltham, MA, USA). Cells were seeded in 6-well plates or 96-well plates and were transfected using jetPEI^®^siRNA/DNA transfection reagent (Polyplus, Strasbourg, France). Proteasome inhibitor MG132 (Beyotime, Shanghai, China) was administered at 20 μM for 4 h.

### 4.4. Western Blot

Tissues and cells were lysed and protein harvested using PIPA buffer (Beyotime, Shanghai, China). Total protein levels were determined by bicinchoninic acid (BCA) analysis (Beyotime, Shanghai, China). Equal amounts of extracts were subjected to SDS-PAGE, transferred to nitrocellulose membranes, and detected with primary antibodies specific for NEDD4L (Cell Signaling Technology, Danvers, MA, USA), or RAC2 (Proteintech Group, Chicago, IL, USA), or BAX (Proteintech Group, Inc. Rosemont, IL, USA), or Bcl-2 (Abcam, Cambridge, UK), or GAPDH (KangChen Bio-Tech, Shanghai, China), and peroxidase-conjugated IgG (Beijing Zhongshan Golden, Beijing, China) with ECL reagent (Bimake, Selleck Chemicals, Shanghai, China).

### 4.5. Co-Immunoprecipitation (IP)

To detect RAC2 ubiquitination, protein was extracted from transfected Caki-1 cells, and immunoprecipitation was performed as described [22]. IPKine heavy-chain-specific secondary antibodies (Abbkine, Wuhan, China) were used to avoid the interference from the antibody light chain.

### 4.6. Gene Expression Analysis

The mRNA levels of *RAC2* and *NEDD4L* in multiple tumors including ccRCC were analyzed using the Tumor Immune Estimation Resource (TIMER) 2.0 database with the Cancer Genome Atlas (TCGA) data (http://timer.cistrome.org/, accessed on 8 March 2024) [65]. The differential expression analysis of *RAC2* and *NEDD4L* in ccRCC tumors and normal tissues, and the correlation of their expression with tumor stage was further performed by Gene Expression Profiling Interactive Analysis (GEPIA2) (http://gepia2.cancer-pku.cn/, accessed on 8 March 2024) [66].

### 4.7. Overall Survival Analysis

The prognostic values of ccRCC for overall survival in 530 patients of *RAC2* and *NEDD4L* were evaluated using the Kaplan–Meier plotter online database (http://kmplot.com/analysis/, accessed on 11 March 2024). Patient samples were divided into two groups according to median expression (high versus low expression) and evaluated using a Kaplan–Meier survival plot, and the log-rank *p* value and hazard ratio (HR) with 95% confidence intervals were calculated.

### 4.8. Correlation of Clinicopathological Features and Genes

The clinicopathological data of 523 patients with ccRCC were downloaded from UCSC Xena (https://xenabrowser.net/, accessed on 14 March 2024). The clinicopathological parameters included age, gender, neoplasm histologic grade, pathologic M, N, T, tumor stage, and vital status. The 523 patients were divided into high and low RAC2 or NEDD4L expression groups according to the median expression value of the genes. The relationship between gene expression levels and clinicopathological parameters was evaluated using chi-squared test.

### 4.9. Significant Prognostic Marker Analysis

The survival data of 523 patients with ccRCC were downloaded from UCSC Xena. Univariate and multivariate Cox regression analysis were performed to identify independent prognostic clinical factors using IBM SPSS statistics 25.

### 4.10. The Relationship Between Gene Expression and Tumor Microenvironment

The tumor sample data in TCGA-KIRC.htseq_FPKM were retained. The content of different types of immune cells in ccRCC tumor tissues was further achieved by “CIBERSORT.R” to quantify the proportion of 22 immune cell types in ccRCC samples with high and low RAC2 or NEDD4L expression. R version 4.1.2 software with the BiocManager, ggplot2, and ggpubr packages were used for analysis and plotting. TIMER 2.0 was used to analyze the relationship between RAC2 and NEDD4L expression and tumor-infiltrating immune cells in ccRCC [65].

### 4.11. Gene Set Enrichment Analysis (GSEA)

The Cancer Genome Atlas (TCGA) gene expression RNASeq (HTSeq-Fragments Per Kilobase per Million (FPKM)) data of ccRCC patients were downloaded from UCSC Xena (http://xena.ucsc.edu/, accessed on 14 March 2024). GSEA 4.2.3 was used to investigate pathways enriched in the high- and low-risk subgroups of RAC2 or NEDD4L. The c2.cp.kegg.v2022.1.Hs.symbols.gmt and c6.all.v2022.1.Hs.symbols.gmt files were chosen as the gene set database. The number of permutations is 1000, and the nominal (NOM) *p*-value < 0.05, false discovery rate (FDR) q-value < 0.1, and normalized enrichment score (NES) > 1 were used to screen statistically significant pathways.

### 4.12. Profiling of Co-Expressed Genes

We used the cBioPortal database (https://www.cbioportal.org/, accessed on 21 March 2024) to screen the co-expressed genes of RAC2 in ccRCC. The top 30 significant genes correlated with RAC2 were selected for further protein and protein interaction networks analysis using STRING (https://cn.string-db.org/, accessed on 21 March 2024). Filter was applied to show only experimentally supported interactions, or curated database, text mining, and co-expression supported interactions, or all supported interactions.

### 4.13. Cell Viability Assay

In vitro cell viability was determined by MTS assay. The 1 × 10^5^/well Caki-1 cells were seeded into 96-well culture plates. At the indicated time points, 20 μL of CellTiter 96^®^ AQueous One Solution Reagent (Promega, Madison, WI, USA) was added to the media for 2 h incubation at 37 °C and the absorbance at 490 nm was measured.

### 4.14. Apoptosis Analysis

Apoptosis was analyzed by fluorescence microscopy using an Annexin V-AbFlourTM 488/Propidium Iodide (PI) apoptosis detection kit (Abbkine, Wuhan, China) according to the manufacturer’s instructions.

### 4.15. Colony-Forming Assay

Caki-1 cells were harvested and dispersed into a suspension of single cells in growth medium. A total of 5000 cells were resuspended in 1 mL growth medium containing 0.35% low melting temperature agarose (Promega, Madison, WI, USA) and were overlaid on 1.5 mL solidified 0.6% low melting temperature agarose in 6-well plates. The number of colonies formed in the soft agar was counted after two weeks.

### 4.16. Transwell Cell Migration

In vitro cell migration assay was performed using Transwell chamber (Merck, Philadelphia, PA, USA) equipped with Matrigel matrix diluted in serum-free medium to a final concentration of 200 μg/mL. A total of 50,000 serum-starved cells were added to serum-free medium in the upper chamber and complete medium with 10% FBS in the lower chamber. After 24 h, cells that had migrated to the underside of the membrane were fixed with 4% paraformaldehyde and stained with 0.1% hematoxylin. To quantify migrated cells on each membrane, the number of cells in 3 randomly selected, non-overlapping fields was counted under the microscope and averaged.

### 4.17. Statistical Analysis

IBM SPSS statistics 25, R version 4.1.2, and RStudio-2022.02.0-443 software were used for statistical analyses. Measurement data are presented as mean ± SE. Independent samples *t*-test and paired samples *t*-test were used to analyze the differential expression levels of *RAC2* and *NEDD4L* mRNA between the ccRCC tissues and the adjacent normal tissues from the TCGA database. Univariate and multivariate analyses were performed using Cox proportional hazards regression model. Statistically significant differences were considered when *p* < 0.05 (* *p* < 0.05, ** *p* < 0.01, *** *p* < 0.001, **** *p* < 0.0001).

## 5. Conclusions

To summarize, we have demonstrated for the first time that RAC2 is a novel target of NEDD4L that may contribute to ccRCC carcinogenesis. Meanwhile, NEDD4L is also the second E3 ligase identified to regulate RAC2 expression. Reduced *NEDD4L* expression, attributed to the upregulation of RAC2 expression, further promoted cell growth and migration, decreased cell apoptosis, and induced pro-tumorigenic immune and multiple tumor-associated pathways, resulting in ccRCC development. Of course, the specific mechanisms of *NEDD4L* in the pathways need to be further elucidated, and other targets of *NEDD4L* may also participate in ccRCC. Taken together, our findings may lead to new diagnostic and therapeutic approaches for ccRCC and provide new insights into the post-translational regulation of *RAC2*.

## Figures and Tables

**Table 1:** Differential gene expression in *NEDD4L* CKO mice.

Accession	Gene	Average TEST	Average CON	Fold_Change	*p*_Value	Type
P19001	*Krt19*	1.191165331	0.768168202	1.550657	0.000597359	Up
P14069	*S100a6*	1.19144703	0.772410035	1.542506	0.017138284	Up
Q60715	*P4ha1*	1.186167092	0.786114744	1.508898	0.025846641	Up
Q80XP8	*Fam76b*	1.170818847	0.795586378	1.471643	0.042585078	Up
P11679	*Krt8*	1.165250956	0.801730113	1.45342	0.003393251	Up
Q05144	*Rac2*	1.157987382	0.803860715	1.440532	0.011042709	Up
Q02496	*Muc1*	1.152986142	0.822520309	1.401772	0.010645896	Up
P50543	*S100a11*	1.141450121	0.833313567	1.369773	0.005128123	Up
Q9DCV7	*Krt7*	1.131805116	0.837824701	1.350885	0.003855372	Up
Q60847	*Col12a1*	1.11728942	0.847447277	1.318418	0.034904913	Up
P05784	*Krt18*	1.118018116	0.849494069	1.316099	0.023242217	Up
Q80YX1	*Tnc*	1.109304778	0.85299358	1.300484	0.002004505	Up
Q9CX80	*Cygb*	1.108984548	0.855302051	1.2966	0.01201333	Up
Q62148	*Aldh1a2*	1.106761812	0.865914924	1.278142	0.021357294	Up
Q9WTK5	*Nfkb2*	1.101540337	0.882545669	1.24814	0.013172931	Up
Q61789	*Lama3*	1.094577663	0.877388797	1.24754	0.029023026	Up
Q03160	*Grb7*	1.100872815	0.882540789	1.24739	0.00511631	Up
Q61398	*Pcolce*	1.100151094	0.886163675	1.241476	0.012409304	Up
A2AE20	*Spaca5*	1.09077901	0.884667883	1.232981	0.048629732	Up
P16110	*Lgals3*	1.089749107	0.896378915	1.215724	0.021688699	Up
P35821	*Ptpn1*	1.081971695	0.897923239	1.204971	0.030206191	Up
O08739	*Ampd3*	1.086732611	0.903548093	1.202739	0.014765244	Up
Q9Z2V4	*Pck1*	0.921913315	1.112294356	0.828839	0.032354526	Down
P97816	*S100g*	0.919517244	1.109824442	0.828525	0.038471214	Down
P35288	*Rab23*	0.903732761	1.093617059	0.82637	0.04218177	Down
Q3TC46	*Patl1*	0.921289116	1.119388176	0.823029	0.008794312	Down
Q8BI72	*Cdkn2aip*	0.911030165	1.110860507	0.820112	0.032722466	Down
P15947	*Klk1*	0.903138588	1.116521088	0.808886	0.013255055	Down
P11152	*Lpl*	0.909904377	1.133845637	0.802494	0.008626671	Down
Q9CPV3	*Mrpl42*	0.899784953	1.123797176	0.800665	0.025629291	Down
P97819	*Pla2g6*	0.882142779	1.14325071	0.771609	0.026488152	Down
Q8R216	*Sirt4*	0.889866204	1.155003037	0.770445	0.019617311	Down
Q6WKZ8	*Ubr2*	0.887824441	1.163670445	0.762952	0.032463371	Down
Q8R0J7	*Vps37b*	0.870487649	1.165277357	0.747022	0.013549489	Down
Q9JKS4	*Ldb3*	0.830937894	1.232832053	0.674007	0.046087213	Down
P48787	*Tnni3*	0.825220095	1.279357586	0.645027	0.034347885	Down
P13542	*Myh8*	0.808632525	1.264221028	0.639629	0.010339535	Down
Q9R062	*Gyg1*	0.829470781	1.32817325	0.62452	0.009931768	Down
O70468	*Mybpc3*	0.759612684	1.27861755	0.594089	0.01629088	Down
P09541	*Myl4*	0.791432574	1.338312631	0.591366	0.014366111	Down
E9Q401	*Ryr2*	0.819324038	1.395412486	0.587155	0.046091099	Down
Q9QVP4	*Myl7*	0.756409591	1.318909268	0.573511	0.022488194	Down
A2ASS6	*Ttn*	0.76398169	1.348053453	0.56673	0.039856283	Down
Q02566	*Myh6*	0.719529118	1.353878057	0.531458	0.023257689	Down
P19123	*Tnnc1*	0.768219584	1.465107903	0.524343	0.046286285	Down
Q8BRJ4	*Ppp1r3e*	0.692546394	1.418836959	0.488109	0.043856029	Down
O09161	*Casq2*	0.694407278	1.435731702	0.483661	0.048301177	Down
Q9JI91	*Actn2*	average TEST	1.521829164	0.428983	0.047167126	Down
P19001	*Krt19*	1.191165331	average CON	1.550657	0.000597359	Down
P14069	*S100a6*	1.19144703	0.768168202	1.542506	0.017138284	Down

**Table 2:** Relationship between *NEDD4L* and *RAC2* expression level and clinicopathological variables in ccRCC patients.

Classification	Total	*NEDD4L* Expression		χ^2^	*p*	Total	*RAC2* Expression		χ^2^	*p*
		High	Low				High	Low		
Age										
≥	278	134	144	0.688	0.407	278	139	139	0.002	0.963
<	245	127	118			245	122	123		
Sex										
Female	180	99	81	2.851	0.091	180	83	97	1.580	0.209
Male	343	162	181			343	178	165		
Grade										
G1/2/X	245	144	101	14.509	<0.001	245	96	149	21.190	<0.001
G3/4	278	117	161			278	165	113		
TNM stage										
Ⅰ/Ⅱ	318	181	137	15.964	<0.001	318	145	173	6.020	0.014
Ⅲ/Ⅳ	205	80	125			205	116	89		
Invasive depth										
T1/2	336	188	148	13.749	<0.001	336	157	179	3.797	0.051
T3/4	187	73	114			187	104	83		
Lymph node metastasis										
N0/X	508	256	252	1.696	0.193	508	248	260	8.348	0.004
N1	15	5	10			15	13	2		
Distant metastasis										
M0/X	446	236	210	10.981	0.001	446	214	232	4.478	0.034
M1	77	25	52			77	47	30		
Status										
Alive	353	199	154	18.182	<0.001	353	160	193	9.107	0.003
Dead	170	62	108			170	101	69		

**Table 3:** Univariate and multivariate analyses of overall survival in TCGA patients with ccRCC using Cox regression model.

Variables	Univariate Analyses		Multivariate Analyses	
	Hazard Ratio (95% CI)	*p* Value	Hazard Ratio (95% CI)	*p* Value
Age (≥60 vs. <60)	1.800 (1.312–2.469)	<0.001	1.539 (1.116–2.122)	0.009
Sex (female vs. male)	1.078 (0.789–1.474)	0.637		
Grade (G3/4 vs. G1/2/X)	2.694 (1.911–3.799)	<0.001	1.682 (1.154–2.452)	0.007
Stage (Ⅲ/Ⅳ vs. Ⅰ/Ⅱ)	3.984 (2.888–5.496)	<0.001	2.552 (1.280–5.087)	0.008
T (T3/4 vs. T1/2)	3.227 (2.373–4.387)	<0.001	0.813 (0.446–1.483)	0.500
N (N1 vs. N0/X)	3.957 (2.142–7.310)	<0.001	2.304 (1.214–4.373)	0.011
M (M1 vs. M0/X)	4.369 (3.194–5.976)	<0.001	2.113 (1.448–3.084)	<0.001
*NEDD4L* (High vs. Low)	0.506 (0.370–0.691)	<0.001	0.839 (0.709–0.993)	0.041
*RAC2* (High vs. Low)	1.442 (1.061–1.958)	0.019	0.924 (0.801–1.067)	0.281

**Table 4:** GSEA pathways upregulated due to high expression of *NEDD4L*.

GS <br> Follow Link to MSigDB	SIZE	NES	NOM *p*-Val	FDR q-Val
KEGG_VALINE_LEUCINE_AND_ISOLEUCINE_DEGRADATION	44	−2.35	<0.001	<0.001
KEGG_PROPANOATE_METABOLISM	32	−2.25	<0.001	<0.001
KEGG_FATTY_ACID_METABOLISM	42	−1.99	<0.001	0.001
KEGG_PEROXISOME	78	−1.94	<0.001	0.002
TBK1.DF_DN	284	−1.92	<0.001	0.002
KEGG_CITRATE_CYCLE_TCA_CYCLE	30	−1.90	0.001	0.003
KEGG_BUTANOATE_METABOLISM	34	−1.80	0.001	0.007
KEGG_PYRUVATE_METABOLISM	40	−1.80	<0.001	0.007
MTOR_UP.N4.V1_DN	176	−1.80	<0.001	0.006
KEGG_TERPENOID_BACKBONE_BIOSYNTHESIS	15	−1.77	0.004	0.009
PGF_UP.V1_UP	187	−1.76	<0.001	0.009
KEGG_STEROID_BIOSYNTHESIS	17	−1.74	0.004	0.011
KEGG_PROXIMAL_TUBULE_BICARBONATE_RECLAMATION	23	−1.72	0.003	0.001
KEGG_LYSINE_DEGRADATION	44	−1.67	0.005	0.021
KEGG_GLYCOSYLPHOSPHATIDYLINOSITOL_GPI_ANCHOR_BIOSYNTHESIS	25	−1.65	0.010	0.025
JAK2_DN.V1_DN	127	−1.62	0.001	0.032
KEGG_ENDOMETRIAL_CANCER	52	−1.62	0.007	0.031
KEGG_INOSITOL_PHOSPHATE_METABOLISM	54	−1.62	0.001	0.031
KEGG_PPAR_SIGNALING_PATHWAY	69	−1.59	0.005	0.038

NES: normalized enrichment score; NOM: normal; FDR: false discovery rate. Gene sets with NOM *p*-value < 0.05 and FDR q-value < 0.2 are considered as significant.

**Table 5:** GSEA pathways upregulated due to low expression of *NEDD4L*.

<br> Follow Link to MSigDB	SIZE	NES	NOM *p*-Val	FDR q-Val
KEGG_CYTOKINE_CYTOKINE_RECEPTOR_INTERACTION	263	2.69	<0.001	<0.001
KEGG_INTESTINAL_IMMUNE_NETWORK_FOR_IGA_PRODUCTION	46	2.65	<0.001	<0.001
KEGG_PRIMARY_IMMUNODEFICIENCY	35	2.46	<0.001	<0.001
KEGG_RIBOSOME	87	2.42	<0.001	<0.001
KEGG_GLYCOSAMINOGLYCAN_BIOSYNTHESIS_CHONDROITIN_SULFATE	22	2.25	<0.001	<0.001
KEGG_PROTEASOME	44	2.12	<0.001	<0.001
KEGG_ECM_RECEPTOR_INTERACTION	84	2.09	<0.001	<0.001
KEGG_CHEMOKINE_SIGNALING_PATHWAY	187	2.05	<0.001	0.001
KEGG_NATURAL_KILLER_CELL_MEDIATED_CYTOTOXICITY	113	1.95	<0.001	0.002
KEGG_NOD_LIKE_RECEPTOR_SIGNALING_PATHWAY	62	1.83	<0.001	0.004
KEGG_ANTIGEN_PROCESSING_AND_PRESENTATION	80	1.80	<0.001	0.005
KEGG_P53_SIGNALING_PATHWAY	68	1.76	<0.001	0.007
KEGG_CELL_ADHESION_MOLECULES_CAMS	131	1.69	<0.001	0.012
KEGG_JAK_STAT_SIGNALING_PATHWAY	155	1.63	<0.001	0.019
KEGG_TOLL_LIKE_RECEPTOR_SIGNALING_PATHWAY	102	1.56	<0.001	0.030
KEGG_FC_GAMMA_R_MEDIATED_PHAGOCYTOSIS	95	1.50	0.018	0.043
KEGG_CELL_CYCLE	124	1.46	<0.001	0.055
KEGG_FOCAL_ADHESION	199	1.44	<0.001	0.057
KEGG_T_CELL_RECEPTOR_SIGNALING_PATHWAY	108	1.40	0.036	0.073
KEGG_LEUKOCYTE_TRANSENDOTHELIAL_MIGRATION	116	1.37	0.012	0.082

NES: normalized enrichment score; NOM: normal; FDR: false discovery rate. Gene sets with NOM *p*-value < 0.05 and FDR q-value < 0.2 are considered as significant.

**Table 6:** GSEA pathways upregulated due to high expression of *RAC2*.

GS <br> Follow Link to MSigDB	SIZE	NES	NOM *p*-Val	FDR q-Val
KEGG_INTESTINAL_IMMUNE_NETWORK_FOR_IGA_PRODUCTION	46	2.62	<0.001	<0.001
KEGG_CYTOKINE_CYTOKINE_RECEPTOR_INTERACTION	263	2.59	<0.001	<0.001
KEGG_PRIMARY_IMMUNODEFICIENCY	35	2.53	<0.001	<0.001
KEGG_ANTIGEN_PROCESSING_AND_PRESENTATION	80	2.52	<0.001	<0.001
KEGG_NATURAL_KILLER_CELL_MEDIATED_CYTOTOXICITY	131	2.50	<0.001	<0.001
KEGG_T_CELL_RECEPTOR_SIGNALING_PATHWAY	108	2.49	<0.001	<0.001
KEGG_CELL_ADHESION_MOLECULES_CAMS	131	2.48	<0.001	<0.001
KEGG_TOLL_LIKE_RECEPTOR_SIGNALING_PATHWAY	102	2.27	<0.001	<0.001
KEGG_B_CELL_RECEPTOR_SIGNALING_PATHWAY	75	2.27	<0.001	<0.001
KEGG_JAK_STAT_SIGNALING_PATHWAY	155	2.07	<0.001	<0.001
KEGG_FC_GAMMA_R_MEDIATED_PHAGOCYTOSIS	95	2.05	<0.001	<0.001
KEGG_LEUKOCYTE_TRANSENDOTHELIAL_MIGRATION	116	1.97	<0.001	<0.001
KEGG_PROTEASOME	44	1.77	0.003	0.004
KEGG_VEGF_SIGNALING_PATHWAY	76	1.71	0.004	0.008
KEGG_APOPTOSIS	87	1.66	0.001	0.011
KEGG_CELL_CYCLE	124	1.63	<0.001	0.014
KEGG_DNA_REPLICATION	36	1.62	0.011	0.017
KEGG_REGULATION_OF_ACTIN_CYTOSKELETON	212	1.59	0.002	0.023
KEGG_P53_SIGNALING_PATHWAY	68	1.54	0.005	0.032
KEGG_LYSOSOME	121	1.50	0.011	0.045

NES: normalized enrichment score; NOM: normal; FDR: false discovery rate. Gene sets with NOM *p*-value < 0.05 and FDR q-value < 0.2 are considered as significant.

## Data Availability

The data sources for this study are based on publicly available databases. For detailed information, please refer to the Materials section of the article. The process analysis data can be obtained from the authors of the article.

## Supplementary Materials

The following supporting information can be downloaded at: https://www.mdpi.com/article/10.3390/ijms252211933/s1.
