# Supplementary material for: NEDD4L Suppresses Proliferation and Promotes Apoptosis by Ubiquitinating RAC2 Expression and Acts as a Prognostic Biomarker in Clear Cell Renal Cell Carcinoma
Source: Int J Mol Sci. 2024 Nov 6;25(22):11933. doi: 10.3390/ijms252211933 (PMC11594477; doi:10.3390/ijms252211933)
Supplement: Supplementary file 1 [file ijms-25-11933-s001.zip › ijms-3282785-supplementary.pdf]

Supplementary Table S1. KEGG enrichment analysis of *NEDD4L* CKO mice

| Category | Term | Count | PValue | Genes | Fold Enrichment |
|---|---|---|---|---|---|
| GOTERM_BP_FAT | GO:0030334~regulation of cell migration | 7 | 5.63E-04 | GRB7, LGALS3, LAMA3, RAC2, TNC, CYGB, S100A11 | 6.01926064 |
| GOTERM_BP_FAT | GO:2000145~regulation of cell motility | 7 | 7.69E-04 | GRB7, LGALS3, LAMA3, RAC2, TNC, CYGB, S100A11 | 5.675454013 |
| GOTERM_BP_FAT | GO:0007155~cell adhesion | 8 | 8.09E-04 | LGALS3, MUC1, KRT18, LAMA3, COL12A1, RAC2, TNC, S100A11 | 4.570485036 |
| GOTERM_BP_FAT | GO:0022610~biological adhesion | 8 | 8.31E-04 | LGALS3, MUC1, KRT18, LAMA3, COL12A1, RAC2, TNC, S100A11 | 4.550760378 |
| GOTERM_BP_FAT | GO:0040012~regulation of locomotion | 7 | 0.001021429 | GRB7, LGALS3, LAMA3, RAC2, TNC, CYGB, S100A11 | 5.377740772 |
| GOTERM_BP_FAT | GO:0051270~regulation of cellular component movement | 7 | 0.001118415 | GRB7, LGALS3, LAMA3, RAC2, TNC, CYGB, S100A11 | 5.285324604 |
| GOTERM_BP_FAT | GO:0060429~epithelium development | 7 | 0.001605217 | LGALS3, KRT19, ALDH1A2, LAMA3, KRT8, TNC, KRT7 | 4.930279898 |
| GOTERM_BP_FAT | GO:0097435~supramolecular fiber organization | 6 | 0.001876787 | KRT19, P4HA1, COL12A1, KRT8, RAC2, KRT7 | 6.130675526 |
| GOTERM_BP_FAT | GO:0097190~apoptotic signaling pathway | 5 | 0.004114876 | LGALS3, PTPN1, MUC1, KRT18, KRT8 | 7.021816337 |
| GOTERM_BP_FAT | GO:0045104~intermediate filament cytoskeleton organization | 3 | 0.004266976 | KRT19, KRT18, KRT7 | 29.13684211 |
| GOTERM_BP_FAT | GO:0045103~intermediate filament-based process | 3 | 0.004355073 | KRT19, KRT18, KRT7 | 28.83333333 |
| GOTERM_BP_FAT | GO:0048646~anatomical structure formation involved in morphogenesis | 6 | 0.008531863 | KRT19, ALDH1A2, LAMA3, COL12A1, KRT8, NFKB2 | 4.308171206 |
| GOTERM_BP_FAT | GO:0048870~cell motility | 7 | 0.009687321 | GRB7, LGALS3, LAMA3, RAC2, TNC, CYGB, S100A11 | 3.437289338 |
| GOTERM_BP_FAT | GO:0051674~localization of cell | 7 | 0.009736656 | GRB7, LGALS3, LAMA3, RAC2, TNC, CYGB, S100A11 | 3.433634592 |
| GOTERM_BP_FAT | GO:0030155~regulation of cell adhesion | 5 | 0.010913993 | LGALS3, MUC1, LAMA3, RAC2, TNC | 5.314900154 |
| GOTERM_BP_FAT | GO:0040011~locomotion | 7 | 0.016993977 | GRB7, LGALS3, LAMA3, RAC2, TNC, CYGB, S100A11 | 3.04941769 |
| GOTERM_BP_FAT | GO:0061564~axon development | 4 | 0.01792758 | LAMA3, S100A6, RAC2, TNC | 6.734793187 |
| GOTERM_BP_FAT | GO:0012501~programmed cell death | 7 | 0.019035705 | LGALS3, PTPN1, MUC1, KRT18, ALDH1A2, KRT8, KRT7 | 2.974973131 |
| GOTERM_BP_FAT | GO:0034097~response to cytokine | 5 | 0.019565271 | PTPN1, KRT18, ALDH1A2, KRT8, NFKB2 | 4.470284238 |
| GOTERM_BP_FAT | GO:0098609~cell-cell adhesion | 5 | 0.019754417 | LGALS3, KRT18, LAMA3, RAC2, S100A11 | 4.457326892 |
| GOTERM_BP_FAT | GO:0007010~cytoskeleton organization | 6 | 0.020422925 | PTPN1, KRT19, KRT18, KRT8, RAC2, KRT7 | 3.473023839 |
| GOTERM_BP_FAT | GO:0016477~cell migration | 6 | 0.021241435 | GRB7, LGALS3, LAMA3, RAC2, CYGB, S100A11 | 3.438509317 |
| GOTERM_BP_FAT | GO:0097284~hepatocyte apoptotic process | 2 | 0.021464979 | KRT18, KRT8 | 87.87301587 |
| GOTERM_BP_FAT | GO:0097191~extrinsic apoptotic signaling pathway | 3 | 0.022972885 | LGALS3, KRT18, KRT8 | 12.08733624 |

| | | | | | |
|---|---|---|---|---|---|
| GOTERM_BP_FAT | GO:0030335~positive regulation of cell migration | 4 | 0.02325047 | GRB7, LGALS3, RAC2, S100A11 | 6.100275482 |
| GOTERM_BP_FAT | GO:0008219~cell death | 7 | 0.025010231 | LGALS3, PTPN1, MUC1, KRT18, ALDH1A2, KRT8, KRT7 | 2.800809483 |
| GOTERM_BP_FAT | GO:0070268~cornification | 2 | 0.025503604 | KRT8, KRT7 | 73.81333333 |
| GOTERM_BP_FAT | GO:2000147~positive regulation of cell motility | 4 | 0.026044754 | GRB7, LGALS3, RAC2, S100A11 | 5.839662447 |
| GOTERM_BP_FAT | GO:0006928~movement of cell or subcellular component | 7 | 0.026902995 | GRB7, LGALS3, LAMA3, RAC2, TNC, CYGB, S100A11 | 2.755403868 |
| GOTERM_BP_FAT | GO:2001234~negative regulation of apoptotic signaling pathway | 3 | 0.027642157 | LGALS3, PTPN1, MUC1 | 10.94071146 |
| GOTERM_BP_FAT | GO:0009790~embryo development | 5 | 0.02765984 | KRT19, ALDH1A2, LAMA3, COL12A1, KRT8 | 4.022086603 |
| GOTERM_BP_FAT | GO:0040017~positive regulation of locomotion | 4 | 0.028116614 | GRB7, LGALS3, RAC2, S100A11 | 5.669226831 |
| GOTERM_BP_FAT | GO:0051272~positive regulation of cellular component movement | 4 | 0.028339775 | GRB7, LGALS3, RAC2, S100A11 | 5.651863196 |
| GOTERM_BP_FAT | GO:0060706~cell differentiation involved in embryonic placenta development | 2 | 0.029526392 | KRT19, KRT8 | 63.63218391 |
| GOTERM_BP_FAT | GO:0048468~cell development | 7 | 0.029979356 | KRT19, ALDH1A2, LAMA3, KRT8, S100A6, RAC2, TNC | 2.688870386 |
| GOTERM_BP_FAT | GO:0032989~cellular component morphogenesis | 5 | 0.031989578 | KRT19, LAMA3, KRT8, S100A6, RAC2 | 3.844444444 |
| GOTERM_BP_FAT | GO:0042127~regulation of cell proliferation | 6 | 0.036699299 | LGALS3, ALDH1A2, S100A6, RAC2, TNC, S100A11 | 2.982758621 |
| GOTERM_BP_FAT | GO:0030036~actin cytoskeleton organization | 4 | 0.040996754 | PTPN1, KRT19, KRT8, RAC2 | 4.888300221 |
| GOTERM_BP_FAT | GO:0007162~negative regulation of cell adhesion | 3 | 0.04344323 | LGALS3, MUC1, TNC | 8.543209877 |
| GOTERM_BP_FAT | GO:0045214~sarcomere organization | 2 | 0.045460334 | KRT19, KRT8 | 41.00740741 |
| GOTERM_BP_FAT | GO:0030855~epithelial cell differentiation | 4 | 0.045622513 | LGALS3, KRT19, KRT8, KRT7 | 4.68358714 |
| GOTERM_BP_FAT | GO:0006915~apoptotic process | 6 | 0.048684529 | LGALS3, PTPN1, MUC1, KRT18, ALDH1A2, KRT8 | 2.763854219 |
| GOTERM_BP_FAT | GO:0035987~endodermal cell differentiation | 2 | 0.049404809 | LAMA3, COL12A1 | 37.65986395 |

Supplementary Table S2. The sequence of the *NEDD4L* siRNA

| **Name** | **Sense (5'-3')**<br>**Antisense (5'-3')** |
|---|---|
| siR-1 | GACAUCUUUGGAGCCAGUGTT<br>CACUGGCUCCAAAGAUGUGUCTT |
| siR-2 | GCGCGUUCAUCAACUGUCATT<br>UGACAGUUGAUGAACGCGCTT |
| Ctrl | UUCUCCGAACGUGUCACGUTT<br>ACGUGACACGUUCGGAGAATT |

Supplementary Table S3. The top 30 genes correlated with *RAC2*

| Correlated Gene | Cytoband | Spearman's Correlation | p-Value | q-Value |
|---|---|---|---|---|
| IL2RG | Xq13.1 | 0.900423834 | 1.85E-194 | 3.71E-190 |
| CORO1A | 16p11.2 | 0.898371294 | 3.17E-192 | 3.18E-188 |
| PTPN7 | 1q32.1 | 0.890618522 | 3.35E-184 | 2.24E-180 |
| WAS | Xp11.23 | 0.878687577 | 5.74E-173 | 2.88E-169 |
| CXCR3 | Xq13.1 | 0.875737533 | 2.26E-170 | 9.07E-167 |
| SASH3 | Xq26.1 | 0.875011279 | 9.62E-170 | 3.21E-166 |
| BATF | 14q24.3 | 0.871351964 | 1.24E-166 | 3.55E-163 |
| CD3E | 11q23.3 | 0.869464246 | 4.57E-165 | 1.15E-161 |
| SIT1 | 9p13.3 | 0.868066229 | 6.38E-164 | 1.42E-160 |
| FMNL1 | 17q21.31 | 0.862222098 | 2.84E-159 | 5.69E-156 |
| SLA2 | 20q11.23 | 0.85912333 | 6.79E-157 | 1.24E-153 |
| CD2 | 1p13.1 | 0.858922808 | 9.63E-157 | 1.61E-153 |
| LCK | 1p35.2 | 0.858656916 | 1.53E-156 | 2.36E-153 |
| CD3D | 11q23.3 | 0.857799114 | 6.77E-156 | 9.70E-153 |
| MAP4K1 | 19q13.2 | 0.856643503 | 4.95E-155 | 6.61E-152 |
| GPSM3 | 6p21.32 | 0.856055808 | 1.35E-154 | 1.69E-151 |
| SIRPG | 20p13 | 0.855454299 | 3.76E-154 | 4.44E-151 |
| SP140 | 2q37.1 | 0.853695767 | 7.31E-153 | 8.14E-150 |
| RHOH | 4p14 | 0.851677113 | 2.10E-151 | 2.21E-148 |
| FERMT3 | 11q13.1 | 0.848487994 | 3.82E-149 | 3.83E-146 |
| DEF6 | 6p21.31 | 0.847112278 | 3.47E-148 | 3.32E-145 |
| TRAF3IP3 | 1q32.2 | 0.846772979 | 5.97E-148 | 5.44E-145 |
| HCST | 19q13.12 | 0.843395435 | 1.22E-145 | 1.05E-142 |
| IL12RB1 | 19p13.11 | 0.843371438 | 1.26E-145 | 1.05E-142 |
| UBASH3A | 21q22.3 | 0.842592987 | 4.22E-145 | 3.39E-142 |
| SELPLG | 12q24.11 | 0.841768565 | 1.51E-144 | 1.16E-141 |
| CD37 | 19q13.33 | 0.84071158 | 7.60E-144 | 5.65E-141 |
| CD27 | 12p13.31 | 0.838020514 | 4.45E-142 | 3.19E-139 |
| SH3BP1 | 22q13.1 | 0.837741147 | 6.77E-142 | 4.68E-139 |
| ITGAL | 16p11.2 | 0.836677779 | 3.30E-141 | 2.21E-138 |

Supplementary Table S4. *RAC2*-related gene enrichment analysis

| **Term description** | **Background gene count** | **Strength** | **False discovery rate** | **Matching proteins** |
|---|---|---|---|---|
| Th1 and Th2 cell differentiation | 85 | 1.57 | 9.18E-05 | CD3D,CD3E,IL2RG,IL12RB1,LCK |
| Th17 cell differentiation | 99 | 1.51 | 9.18E-05 | CD3D,CD3E,IL2RG,IL12RB1,LCK |
| Primary immunodeficiency | 37 | 1.84 | 9.18E-05 | CD3D,CD3E,IL2RG,LCK |
| Hematopoietic cell lineage | 90 | 1.45 | 0.001 | CD3D,CD37,CD3E,CD2 |
| PD-L1 expression and PD-1 checkpoint pathway in cancer | 87 | 1.47 | 0.001 | BATF,CD3D,CD3E,LCK |
| Human T-cell leukemia virus 1 infection | 210 | 1.18 | 0.0011 | CD3D,ITGAL,CD3E,IL2RG,LCK |
| Natural killer cell mediated cytotoxicity | 120 | 1.33 | 0.002 | HCST,RAC2,ITGAL,LCK |
| T cell receptor signaling pathway | 100 | 1.28 | 0.0235 | CD3D,CD3E,LCK |
| Leukocyte transendothelial migration | 111 | 1.23 | 0.028 | RAC2,ITGAL,RHOH |
| Cytokine-cytokine receptor interaction | 282 | 0.95 | 0.0336 | CD27,CXCR3,IL2RG,IL12RB1 |
| Yersinia infection | 124 | 1.19 | 0.0336 | RAC2,WAS,LCK |
| Cell adhesion molecules | 138 | 1.14 | 0.0381 | SELPLG,ITGAL,CD2 |
